# A Brief Review on High‐Performance Capacitive Deionization Enabled by Intercalation Electrodes

**DOI:** 10.1002/gch2.202000054

**Published:** 2020-11-05

**Authors:** Zhenzhen Liu, Xu Shang, Haibo Li, Yong Liu

**Affiliations:** ^1^ Ningxia Key Laboratory of Photovoltaic Materials Ningxia University Yinchuan Ningxia 750021 P. R. China; ^2^ School of Materials Science and Engineering Qingdao University of Science and Technology Qingdao Shandong 266042 P. R. China

**Keywords:** capacitance deionization, ion intercalation materials, redox reactions

## Abstract

Owing to the advantages of cost‐effectiveness, environmental‐friendliness and high desalination capacity, capacitive deionization (CDI) has emerged as an advanced desalination technique. Recently, the ions intercalation materials inspired by sodium ion batteries have been widely implemented in CDI due to their exceptional salt removal capacity. They are able to extract sodium ions from the brine through intercalation or redox reactions, instead of electrostatic forces associated with the carbonaceous electrode. As a result, the ions intercalation materials have caught the attention of the CDI research community. In this article, the recent progress in various sodium ion intercalation materials as highly‐efficient CDI electrodes is summarized and reviewed. Further, an outlook on the future development of ion intercalation electrodes is proposed.

## Graphical abstract

At present, intercalation materials have received extensive attention in the field of capacitive deionization (CDI). Herein, the recent progress in sodium ions intercalation materials for CDI‐related applications is discussed and a general summary of their performance and advantages brought by their characteristics is provided.

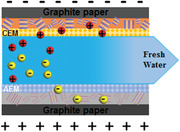

## 1 Introduction

With the rapid population growth and economic development, the requirements for clean drinking water have grown tremendously, while the water consumption around the globe has increased at a rate of 4–8% per year in the last few decades. Meanwhile, a sharply growing amount of freshwater sources is polluted during the process of modelization, which would inevitably aggravate the global water crisis.^[^ ^1^, ^2^ ^]^ To face the challenge, great attempts have been devoted to explore efficient water treatment technologies, among which the desalination is envisioned to be the most feasible approach. Based on the working mechanism, the desalination technologies are generally divided into three broad categories: physical‐, chemical‐, and membrane‐driven desalination techniques. Among these techniques, distillation emerges as the earliest commercial physical desalination technique. It is basically triggered by heat through physical methods and thereby fresh water is separated from nonvolatile solutes by evaporation and subsequent condensation.^[^ ^3^, ^4^ ^]^ The required energy largely depends on the latent heat of evaporation and the degree of heat recovery that can be achieved. The latent heat of water evaporation is two orders of magnitude larger than the minimum separation energy of thermodynamics, which makes thermal desalination technology inherently energy‐intensive by compared to that of pressure‐driven desalination.^[^ ^5^, ^6^, ^7^ ^]^ Nevertheless, aiming to deslinate the highly‐salty feed, heat‐driven desalination is often required. Moreover, various improved versions of the distillation‐based desalination have been developed including multistage flash distillation (MSF) and multieffect distillation (MED).^[^ ^8^, ^9^ ^]^ In order to offset the huge latent heat of evaporation, recent heat‐driven efforts have shifted to the use of renewable energy sources, i.e., solar energy. The optimization of solar thermal desalination system and the application of solar heating membrane distillation (MD) have become popular in this research domain.^[^ ^10^, ^11^, ^12^, ^13^, ^14^ ^]^ However, the distillation‐based techniques are plagued by several obvious issues, such as high energy consumption, corrosion risks, high capital/operational costs, etc.^[^ ^15^, ^16^ ^]^ Membrane‐based desalination technologies (e.g., electrodialysis (ED)^[^ ^17^ ^]^ and reverse osmosis (RO)^[^ ^18^, ^19^ ^]^), have rapidly developed into the most popular technologies in the desalination community due to their advantages of low energy consumption, easy regeneration, and high water quality. Among them, RO is the most widely employed and energy‐saving desalination technique currently. It is based on the semipermeable membrane to selectively pass water molecules forced by pressure.^[^ ^20^, ^21^, ^22^ ^]^ Ensentially, the application of RO accounts for 72% of the global seawater desalination capacity.^[^ ^23^ ^]^ It is largely benefited from the high energy efficiency, which is achieved through high permeability and water‐salt selective thin‐film composite (TFC) polyamide membranes.^[^ ^3^, ^24^ ^]^ Furthermore, in order to allow water to pass through the membrane and thus produce purified water, RO requires excessive hydraulic pressure to generate the osmotic pressure of brine.^[^ ^25^, ^26^ ^]^ Unfortunately, current RO membranes and corresponding modules suffer from the mechanical stability that required to overcome the osmotic pressure encountered during the treatment of high salty solutions. Despite the utilization of TFC polyamide membranes represents a breakthrough in desalination technology in the past dacate, inherent materials have limited further improvements in performance.^[^ ^27^, ^28^ ^]^ Meanwhile, one major drawback of the membrane‐based desalination techniques is the regular replacement of the membrane component due to scaling or fouling, which would inevitably increase their operational and maintenance costs.

Compared with the above dealination technologies, electrochemical‐assisted desalination technique is the most effective when desalinate the brackish water and low‐salinity water. The obvious advantage is that avoid the use of either high pressure or high temperature. Capacitive deionization (CDI), as typical electrochemical desalination techniques, has become a new star in the desalination community due to its merits of high efficiency, low energy consumption and environmental‐benign.^[^ ^29^, ^30^ ^]^ **Table** **1** summarizes the energy consumption of various desalination technologies which firmly confirms the advances of CDI. Actually, CDI is not only employed to achieve seawater desalination and ion separation, but also be applicable for manufacturing, agriculture and mining, which is thereby expected to be a cheap and sustainable technology.^[^ ^31^, ^32^, ^33^ ^]^ The basic principle of CDI is that the ions in the feed solution would migrate to the oppositely‐charged electrodes through the electrostatic force by imposing a low direct voltage (<2.0 V), and stored within the EDL formed on the interface between the electrode and solution. After that, the ions could be released (or enriched) into an alternative water stream by simply removed or reversed the voltage, leading to the regeneration of electrode.^[^ ^34^ ^]^ As compared to traditional desalination techniques, CDI exhibits remarkable advantages: i) Low energy consumption and high water recovery: the voltage across the electrodes of the CDI is below 2.0 V, indicating the only 0.6 KWh∙m^−3^ of electrical energy is required which is significantly lower than that of ED and RO. Meanwhile, the water recovery of CDI can be reached as high as 85%, which is obviously higher than those of traditional desalination techniques (i.e., RO: ≈50%)^[^ ^35^, ^36^, ^37^, ^38^ ^]^; ii) Environmental tolerance: differing from the RO and ED, the regeneration of CDI electrode does not require any additional chemicals, which could effectively reduce the risk of secondary pollution;^[^ ^39^, ^40^ ^]^ iii) Facile operation and minimum maintenance: the CDI device is easy to operate and requires minimum maintenance due to the low scaling or fouling risks. Most importantly, none expensive membrane is involved in CDI which would not bring any extra maintenance cost during the operation;^[^ ^41^, ^42^ ^]^ iv) Highly‐efficient energy recovery: the required voltage of CDI is much lower than other electro‐driven desalination techniques.^[^ ^43^, ^44^ ^]^ On the other hand, the CDI could be directly supplied by some new energy sources, such as biomass energy and solar energy. Moreover, the electric energy released during the desorption process can be captured simultaneously and forwarded to the subsequent cycling.^[^ ^45^ ^]^ Despite many advances of CDI, challenges are remaining, such as parasitic reactions and low desalination capabilities, which have limitted the practical application and further development of CDI. Actually, the CDI is effective for groundwater restoration, brackish water desalination and industrial wastewater regeneration. When dealing with highly‐concentrated brine, the energy consumption would be increased, accompanied by the decrease of energy efficiency and salt removal capacity. Significantly, as an electricity‐based desalination technique, the energy consumption related to CDI has an almost linear relationship with the amount of salt removal. When attempting to reduce energy consumption by introducing a low cell voltage, it would result in a very slow desalination rate and a longer operating durations. The side reactions taking place on CDI electrodes mainly include anodic oxidation reactions, cathodic reduction reactions, etc. The occurrence of side reactions would change the potential of zero charge (PZC) of the electrodes, causing an imbalanced potential distribution across the positive and negative electrodes, which would accelerate the water decomposition, and eventually sabotage the desalination performance of the CDI device. Besides, the side reactions also affect the pH value of the desalinated water (especially during long‐term operations), leading to unsatisfied water quality. Most importantly, the intrinsic characteristics of the carbon materials (i.e., complex porous structure, inherent structural defects, self‐adsorption, wettability limitations and adverse effects of side reactions), have resulted in insufficient desalination capacity of the carbon‐based CDI. To address this issue, various cell architectures have been developed, involving membrane CDI (MCDI),^[^ ^46^ ^]^ flow‐through CDI (FTE‐CDI),^[^ ^47^ ^]^ CDI with flowable electrodes (FCDI), and hybrid CDI (HCDI),^[^ ^48^ ^]^ etc. Among these cell architectures, HCDI enabled by sodium ion intercalation (SII) electrode material has demonstrated ultra‐high desalination capacity due to their superior desalination mechanisms (insertion or redox).

**Table 1:** Summary of the energy consumption of various desalination processes

Technology	Electrode materials	Energy consumption	Energy recovery [%]	Permeate flux [m^3^ per day]	Ref.
RO	TFC cross‐linked fully aromatic polyamide spiral wound	4.35 KWh m^−3^		19.7–24.6	^[^ ^26^ ^]^
	Asymmetric cellulose tri‐acetate hollow fiber	5.00 KWh m^−3^		60.0–67.0	^[^ ^27^ ^]^
MSF		21–58 KWh m^−3^			^[^ ^8^ ^]^
MED		5–6.5 KWh m^−3^			^[^ ^9^ ^]^
CDI	Fee(CN)_6_(PB)	6.67 KT per ion			^[^ ^54^ ^]^
	Na_4_Ti_9_O_20_	0.127 Wh g^−1^	>30		^[^ ^55^ ^]^
	Na_0.71_CoO_2_	34.9 Wh m^−3^	57		^[^ ^58^ ^]^
	PB/PANI composite	0.35 KWh Kg^−1^	74.7		^[^ ^60^ ^]^
	MnHCF	42.6 KT per ion			^[^ ^61^ ^]^
	ZnFe_2_O_4_	0.76 KWh Kg^−1^	23.3		^[^ ^78^ ^]^
	P‐NMO	15.25 µmol J^−1^			^[^ ^82^ ^]^
	Na_0.44_MnO_2_	0.46 KWh Kg^−1^			^[^ ^85^ ^]^
	CuFe@NiFe Prussian Blue	0.376 KWh Kg^−1^			^[^ ^93^ ^]^
	Ti_3_C_2_T*_x_* MXene	0.24 KWh Kg^−1^	5.44		^[^ ^101^ ^]^

**Figure 1:**
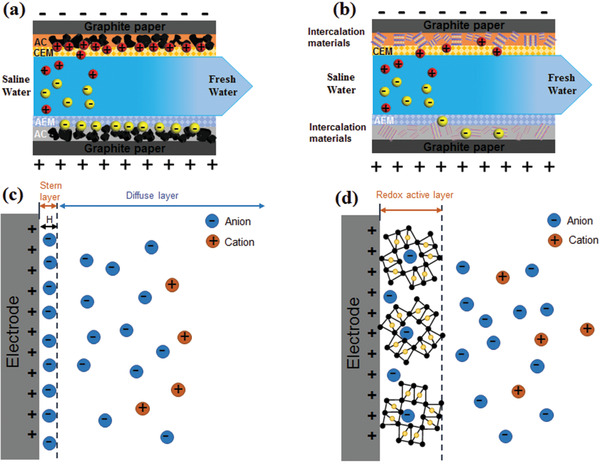
Schematics of CDI process based on a) EDL ion storage and b) ion intercalation storage, the working mechanism of c) EDL ion storage, and d) ion intercalation storage.

With the continuous development of CDI, there are two main ion storage mechanisms, namely, electrical double layer (EDL) ion storage and ion intercalation storage. **Figure** **1**a draws the CDI process based on EDL mechanim. Basically, when a direct voltage difference is imposed on the two oppositely‐positioned electrodes, cations and anions are separately forced toward the negative and positive electrode and hold by EDL, leading to the removal of salty ions. Afterwards, the electrode can be regenerated by either short‐circuiting or applying an reverse voltage. Acturally, the EDL ion storage is the most important electrochemical process for desalination. After a potential difference is applied, ions are electrostatically trapped and capacitively stored in the diffusion layer formed in the pores of the carbon electrode. As shown in Figure 1c, under the combined action of electrochemical potential and electric field force, the anions and cations separate and move to the positive and negative electrodes in the solution, respectively. The ions are arranged on the side of the electrode/solution interface at a certain distance between the electrodes to form a dense EDL, which creates an internal electric field to balance the external electric field. The Gouy–Chapman–Stern model (GCS model) is proposed to explain the EDL ion adsorption.^[^ ^49^ ^]^ When the electrodes interacting with charged species, the EDLs are generated which composed of a dense layer (Helmholtz layer or Stern layer) and a diffusion layer. Therefore, the capacitance value can be expressed by the following formula

(1) $$\frac{1}{C_{dl}}=\frac{1}{C_{st}}+\frac{1}{C_{d}}$$

(2) $$C_{st}=\frac{\varepsilon }{4\pi d}$$

(3) $$C_{d}=\frac{\varepsilon }{\lambda d}\cdot \cosh\left(\frac{Z\cdot \Delta \varphi d}{2}\right)$$

Where *C*_dl_ is the EDL capacitance, *C*_st_ is the Stern layer capacitance, and *C*_d_ is the diffusion layer capacitance. ε is the dielectric constant in the solution, *d* is the thickness of the Stern layer, Δϕ_d_ is the voltage difference of the diffusion layer, λ_d_ is the Debye length, and *Z* is the number of ion charges. In order to improve the storage performance of EDL ions adsorption, researchers have invested massive energy to focusing on electrode materials with high adsorption capacity and sustainable ability. These characteristics are usually related to materials with high specific surface area, resonable pore size distribution, remarkable electrical conductivity and excellent chemical innert.^[^ ^50^ ^]^

Despite the ion storage mechanism based on the principle of EDL is the most common phenomenon in CDI, recent outcomes have figured out that ion intercalation mechanism is more conducive to improve the desalination capacity. As shown in Figure 1b, it involves the utilization of intercalation electrodes (mainly containing transition metals or conductive polymers) to collect ions from brine. The ion storage mechanism involves trapping ions to the crystallographic location of the intercalation electrodes. As shown in Figure 1d, under the action of an external electric field, the ions are attracted to the electrode/solution interface, and then enter the bulk of intercalation electrode. This process is fundamentally different from the EDL charging process that occurs in traditional carbon electrodes. Depending on the volume expansion and structural reversibility of the intercalation material, wider gap sites will produce higher cycle stability, greater ion storage capacity (higher charge storage capacity) and faster kinetics (i.e., the diffusion of ions in the host material).^[^ ^32^, ^51^, ^52^ ^]^ These are due to that such electrodes have been expected to achieve significant salt adsorption capacity and definetly the highly ion selectivity.

In 2014, Lee et al. pioneered the asymmetrical HCDI configuration that utilizes Na_4_Mn_9_O_18_ as the SII electrode (negative electrode) and AC as the chloride ion adsorption electrode (positive electrode).^[^ ^48^ ^]^ During the desalination process, sodium ions are chemically inserted into the NMO electrode instead of being captured by the EDL, while chloride ions are stored in the EDL formed on the surface of the AC electrode. In these “battery” materials, the ions can be chemically stored within their lattice, which is fundamentally different from the electrosorption process that occurs at the surface of the carbon electrodes. As a result, the HCDI system has demonstrated a high desalting capacity of 31.2 mg g^−1^ at 1.2 V in a 580 mg L^−1^ NaCl solution, which has realized significant improvement compared to that of the traditional CDI system (17.1 mg g^−1^).^[^ ^53^ ^]^ Since then, various SII electrodes were proposed, many of which were initially explored in the context of energy storage, such as FeFe(CN)_6_(PB),^[^ ^54^ ^]^ Na_4_Ti_9_O_20_ (NTO),^[^ ^55^ ^]^ NaTi_2_(PO_4_)_3_(NTP),^[^ ^56^ ^]^ Na_3_V_2_(PO_4_)_3_ (NVP),^[^ ^57^ ^]^ Na_0.71_CoO_2_ (NCO),^[^ ^58^ ^]^ Na_0.7_MnO_2_ (NMO),^[^ ^59^ ^]^ polyaniline (PANI)‐tube‐decorated with Prussian blue (PB) nanocrystals (PB/PANI composite),^[^ ^60^ ^]^ MnHCF,^[^ ^61^ ^]^ MoS_2_/rGO^[^ ^62^ ^]^ and Ti_3_C_2_T*_x_*‐Mxene^[^ ^63^ ^]^ etc.

Owing to the superior desalination capacity, the SII materials have attracted great interest in the CDI community, while the desalination capacities of these materials are greatly improved. Meanwhile, the sodium intercalation electrodes are expected to offer high charge efficiencies since the side reactions (such as anode corrosion in CDI) can be effectively restricted. Moreover, extensive studies have featured that the SII based CDI demonstrates the impressive charge storage capacity and energy‐saving desalination capacity in both low and high‐concentrated stream. The emergence of SII materials has greatly expanded the application range of CDI, which can not only be used for desalination, but also be extended to do water repair applications, such as the removal of heavy metals,^[^ ^64^, ^65^ ^]^ valuable elements (such as: lithium),^[^ ^66^, ^67^ ^]^ ion separation,^[^ ^68^, ^69^ ^]^ and water disinfection,^[^ ^70^ ^]^ etc.

This review mainly highlights the recent progress of SII materials for CDI‐related applications and provides a general understanding of their performance and advantages brought by their characteristics. Beyond that, the challenges and prospects of the SII electrodes are discussed as well.

## 2 Sodium Ions Intercalation Materials

In intercalation materials‐based CDI systems, sodium ions are inserted into specific (nonspecific) interstitial positions or atomic planes of the electrode material. Therefore, an ideal intercalation electrode material is expected to have large ion storage capacity (to ensure sufficient desalaintion capacity), large gap sites (to reinforce cyclic stability) as well as fast ion storage kinetics (to promote ion duffusion). Typically, SII exhibits high crystallinity and ordered structrue. In terms of the intercalation ability, most SII are capable of affording the intercalation of cations, such as Li^+^, Na^+^, K^+^, Mg^2+^, and Ca^2+^, etc., while a few SII, such as Mxenes, enable to be inserted by both anions and cations instantaneously. To classify the SII, various methods have been suggested, depending on the dimensions (1D, 2D, and 3D) and the characteristics of the crystal structure. In this article, the SII is discussed by chemical compositions, that is, transition metal oxides (TMO or NaTMO, TM = Mn, Ti, Fe, Ni, Co, etc.), iron pyrophosphate, prussian blue analogues, transition metal dichalcogenides and MXene. TMO have been widely investigated in sodium ion removal due to their diverse structures and easy preparation. Both iron pyrophosphate and prussian blue analogues have an open frame structure, providing abundant directions to accommodate ions. Meanwhile, they have large space for ion adjustment, which is suitable for the adsorption and separation of multivalent ions. Moreover, TMD and MXene are subjected to insert both cations and anions.

### 2.1 Transition Metal Oxides

Benifiting from the highly stability, tunnelable redox potential, abundant active site and variety, the TMO, including MnO_2_, TiO_2_, V_2_O_5_, Na*_x_*Mn*_y_*O*_z_* (NMO), Na*_x_*Co*_y_*O*_z_* (NCO) and Na*_x_*Ti*_y_*O*_z_* (NTO) etc., are extensively studied as highly‐efficient electrode material for CDI.

Titanium dioxide (TiO_2_), as a typical pseudocapacitive material (Ti^4+^/Ti^3+^ in neutral solution), exihbits various advatages such as large capacity, rich resources, nontoxicity, good stability, and environmental friendliness,^[^ ^71^ ^]^ which make TiO_2_ an excellent candidate for CDI. The TiO_2_ crystals exists in three natural form including anatase, brookite and rutile and several synthetic forms (such as TiO_2_ (B) (monoclinic) and TiO_2_ (H) (square), etc., relating to the different spatial arrangement of TiO_6_ octahedron). Previous studies have demonstrated that the the crystal structure of TiO_2_ has a significant impact on the electrochemical/desalination behavior of TiO_2_. For example, Yang et al. have carried out a comparative study on the relationships between CDI performance and crystal structure of TiO_2_ (anatase and rutile). The results manifested that the anatase TiO_2_ structure is more favorable as electrode material for CDI, exhibiting a much higher desalination capacity (41.8 mg g^−1^ in 500 mg L^−1^ NaCl solution at 10 mA g^−1^) than that of the counterpart TiO_2_ electrode (rutile, 28.8 mg g^−1^). ^[^ ^72^ ^]^

**Figure 2:**
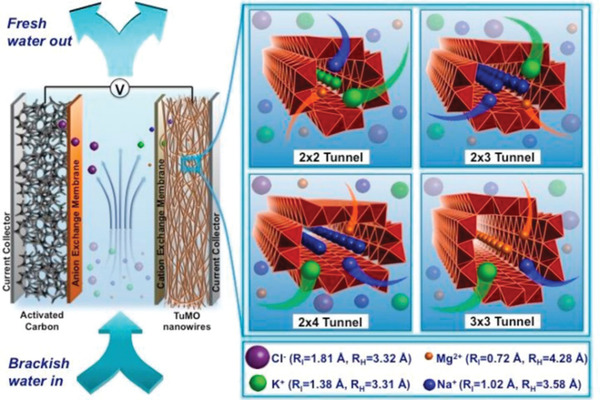
Schematic illustration of ion removal from water via a HCDI AC ‖ TuMOs nanowires device. Reproduced with permission.^[^ ^74^ ^]^ Copyright 2018, Elsevier.

MnO_2_ is viewed as a low‐cost and environmental‐friendly electrodes’ material possessing high electrochemical activity due to the presence of active Mn^3+^/Mn^4+^ redox pair.^[^ ^73^ ^]^ Among more than 30 polymorphisms of MnO_2_, tunnel structured manganese oxides (TuMOs), constructed from MnO_6_ octahedrons with various sizes and shapes, is known to have superior desalination performance. Moreover, the large tunnels in TuMOs contain positively charged cations, which could stabilize the crystal structure. Previous studies have concluded that open tunnel structures associating with TuMOs could offer excellent cation exchange properties. As a result, it would be favorable to energy storage devices. In 2018, Byles et al.^[^ ^74^ ^]^ systematicly investigated the influence of the TuMOs’ tunnel sizes and crystal structure on their desalination performance as electrode for HCDI, as shown in **Figure** **2**. Four types of TuMOs are investigated as HCDI electrodes, namely α‐MnO_2_ (2 × 2 tunnels), manganese oxide with todorokite crystal structure (Tod‐MnO_2_, 3xn tunnels), 2xn‐MnO_2_ and hybrid‐MnO_2_ (combination of 2xn and 3xn tunnels). After evaluation, the desaliatnion capacities of α‐MnO_2_, Tod‐MnO_2_, 2xn MnO_2_, and Hybrid‐MnO2‐based HCDI are 22.1, 23.3, 27.8, and 27.3 mg g^−1^, respectively.

**Figure 3:**
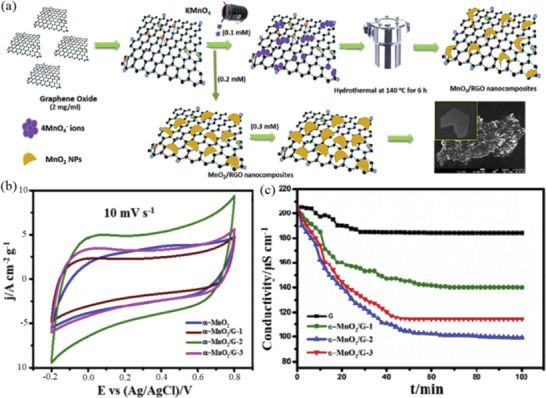
a) Illustration of the nucleation growth mechanism of MnO_2_/G nanocomposites, b) the comparison on CVs among α‐MnO_2_, α‐MnO_2_/G‐1, α‐MnO_2_/G‐2, and α‐MnO_2_/G‐3 at 10 mV s^−1^, c) plots of conductivity versus time. Reproduced with permission.^[^ ^75^ ^]^ Copyright 2020, Elsevier.

Despite the high chemical stability, superior specific capacitance and high density of active surfaces of TMOs electrodes, the electrical conductivity is unsatisfied. To address this issue, Jaoude et al. proposed a simple hydrothermal strategy to realize the controlled synthesis α‐MnO_2_ on the surface of graphene (G), as shown in **Figure** **3**a.^[^ ^75^ ^]^ In this configuration, Na^+^ are inserted into the α‐MnO_2_/G electrode by electrochemical reaction, while Cl^−^ is adsorbed into the graphene electrode. As illustrated in Figure 3b, the α‐MnO_2_/G‐2 demonstrates the highest specific capacity of up to 375 F g^−1^ among all samples, while the G//α‐MnO_2_/G‐2‐based HCDI system exhibits the highest salt removal capacity of 29.5 mg g^−1^ at 1.2 V due to the maximum synergistic effect between G and high‐crystalline α‐MnO_2_ (the neat G sheets mainly contribute to electrical conductivity and prevent the agglomeration of individual nanoparticles).

**Figure 4:**
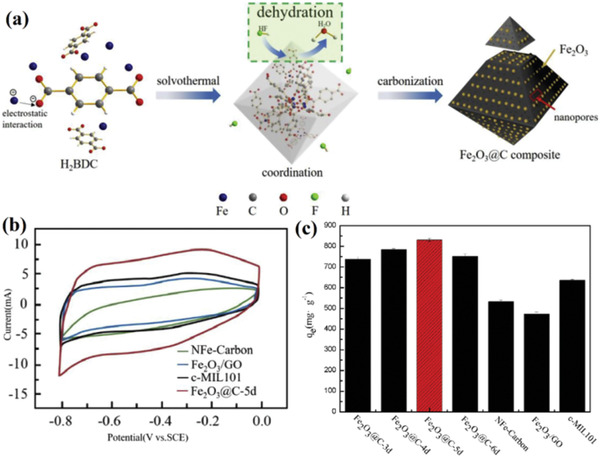
a) Schematic illustration of the HF‐inducing fabrication process of Fe_2_O_3_@C, b) CV curve of Fe_2_O_3_@C‐5d, NFe‐Carbon, c‐MIL101, Fe_2_O_3_/GO, c) salt removal capacity of various electrodes. Reproduced with permission.^[^ ^76^ ^]^ Copyright 2019, Elsevier.

It is firmly believed that the desalination performance of TMOs/C nanocomposite is depending on the dispersion and particle size of TMOs. However, the weak interaction between the TMOs and carbon and inherently non‐uniform distribution in the carbon matrix could results in severe particle aggregation. Luo et al. proposed a facile approach to prepare homogeneous Fe_2_O_3_@C composites via solvothermal carbonization by employing terephthalic acid and ferric chloride as raw materials.^[^ ^76^ ^]^ It is found that most Fe^3+^ are captured by H_2_BDC through electrostatic action and then coordinated with a large number of carboxyl groups. This strong synergy ensures that these metal nodes are evenly distributed in the organic framework, making Fe_2_O_3_ uniformly present in the carbonized matrix. The results have demonstrated that the Fe_2_O_3_@C‐5d displays a high specific capacitance of 115.6 F g^−1^, which is significantly higher that the c‐MIL101 (64.2 F g^−1^), Fe_2_O_3_/GO (50.3 F g^−1^), and NFe‐Carbon (38.8 F g^−1^) due to the uniform distribution of Fe_2_O_3_ as well as the superior pseudocapacitance of Fe_2_O_3_. Furthermore, all as‐prepared composite are employed as electrode of HCDI to remove Pb^2+^. As shown in **Figure** **4**c. it is explored that the Fe_2_O_3_@C‐5d possess the highest Pb^2+^ adsorption capacity of 830.17 mg g^−1^ in 500 mg L^−1^ Pb(NO_3_)_2_ solution, which further evidenced that the well‐dispersion of Fe_2_O_3_ in the carbon matrixes has a significant impact on its desalination performance.

**Figure 5:**
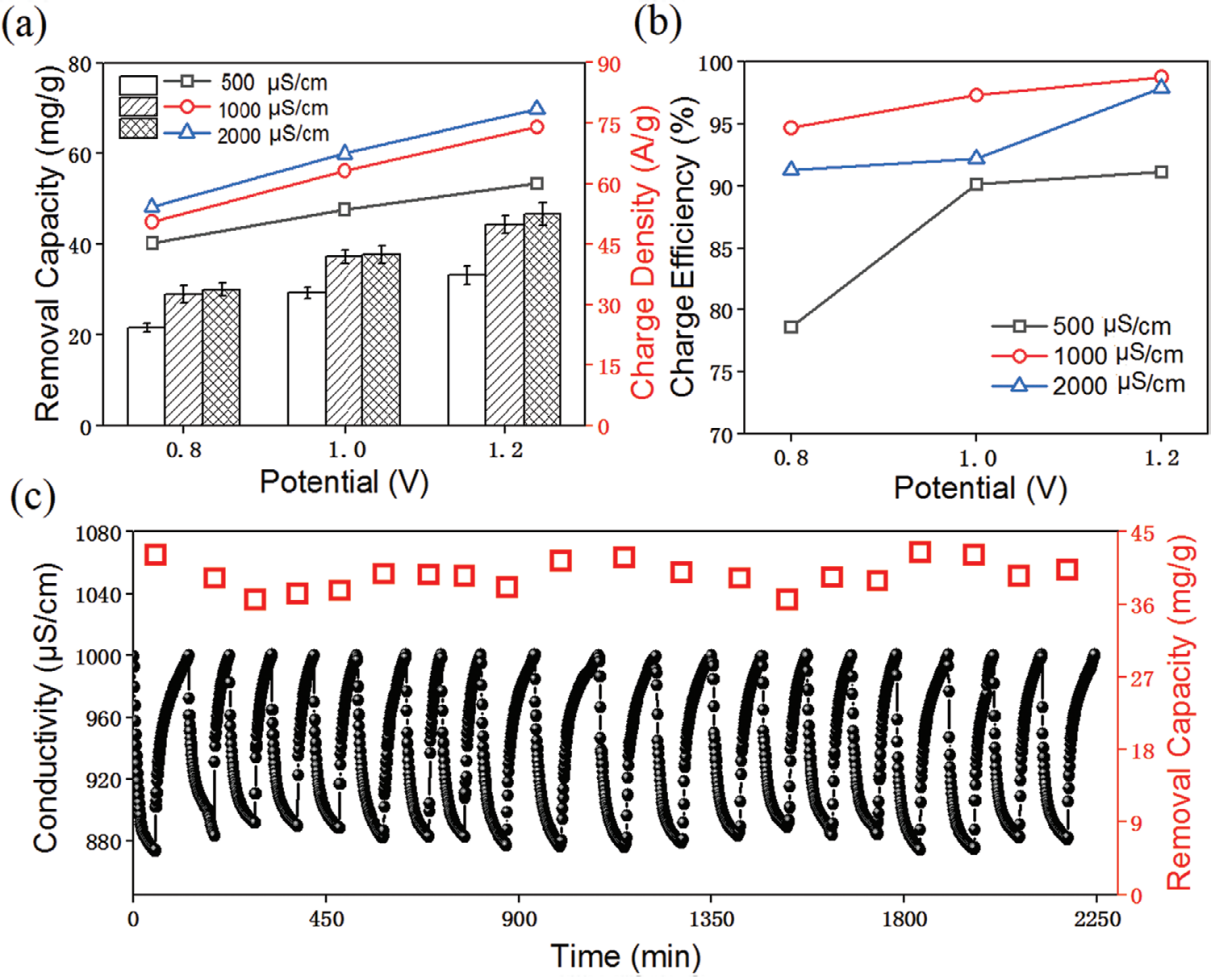
a) Salt removal capacity (mg g^−1^) and current density (A g^−1^) with respect to cell voltage (V), b) charge efficiency, and c) cycling performance of NiCo_2_O_4_ at 1.2 V. Reproduced with permission.^[^ ^79^ ^]^ Copyright 2020, The Royal Society of Chemistry.

Beside TMOs, the binary metal oxides (BMOs) have also attracted great attentions due to their good electronic conductivity, low diffusion resistance and high theoretical capacity.^[^ ^77^ ^]^ For instance, ferrites are deemed as protential electrode candidate for CDI originating from its high chemical stability, abundance in nature, low manufacturing cost, as well as their tunable of the composition. Yu et al. synthesized well‐crystallized zinc spinel ferrite (ZFO) nanoparticles and utilized as electrode material for HCDI.^[^ ^78^ ^]^ The ZFO‐based HCDI exihbits ultrahigh desalination capacity of 136.6 mg g^−1^ at current density of 30 mA g^−1^ and initial brine concentration of 100 × 10^−3^ m and superior cycling performance (no obvious decilination in 200 cycles). Laterly, Li et al. synthesized hollowed sea urchin‐like NiCo_2_O_4_ nanocrystals as cathode for HCDI.^[^ ^79^ ^]^ As verified in **Figure** **5**a,b, the NiCo_2_O_4_ based HCDI system possess high salt removal capacity of 44.3 mg g^−1^ and charge efficiency of 98.7%. Meanwhile, the selective removal performance of the NiCo_2_O_4_ based HCDI between various cations (K^+^, Na^+^, and Mg^2+^) are also investigated. The results imply that K^+^ is more easily inserted into the crystal layer of NiCo_2_O_4_, while the ionic radius has priority (K^+^ (331 pm) < Na^+^ (358 pm) < Mg^2+^ (428 pm)). Beyond that, the salt removal capacity as high as 42.9 mg g^−1^ can be achieved for the NiCo_2_O_4_ based HCDI after 20 cycles (≈97.5% capacity retention) in Figure 5c, suggesting an excellent longterm stability of the device.

### 2.2 Sodium Transition Metal Oxides

**Figure 6:**
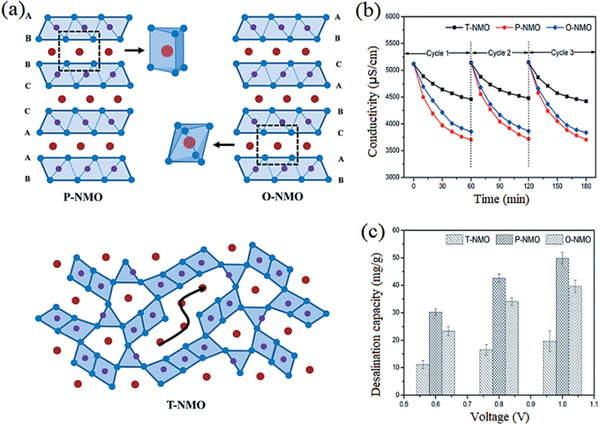
a) Crystal structures of T‐NMO, P‐NMO, and O‐NMO, b) effluent conductivity of various crystallographic NMOs within three representative cycles at 1.0 V, c) comparison of the desalination capacity among various crystallographic NMOs at different voltages. Reproduced with permission.^[^ ^82^ ^]^ Copyright 2019, The Royal Society of Chemistry.

Sodium manganese oxide (Na*_x_*MnO_2_, NMO, 0 < *x *≤ 1) exists in multiple crystal forms, i.e., 2D layered structure (0.34 < *x* ≤ 1) and 3D tunnel structure (*x* ≤ 0.44).^[^ ^80^, ^81^ ^]^ Importantly, the sodium ions diffusion and storage behavior is highly denpendent upon the structure of Na*_x_*MnO_2_. In order to clarify the influence of the NMO's crystal structure on their desalination performance, Lv et al. prepared NMO with various structures (P2 group (P‐NMO) as tunnel‐type NMO (T‐NMO) and O3 group (O‐NMO) as layered NMO) and used as electrode material for HCDI.^[^ ^82^ ^]^ In **Figure** **6**, it is found that the salt removal capacity of NMO electrodes follows the order of P‐NMO> O‐NMO> T‐NMO, since the T‐NMO possesses the lowest desalination capacity due to its limited theoretical capacity for reversible Na^+^ intercalation/deintercalation. In terms of O‐NMO, the Na^+^accommodation at the octahedral site leads to a higher direct conduction activation energy of Na^+^ between adjacent octahedral sites. Further, Na^+^ would overcome the diffusion barrier by migrating to the tetrahedral interstitial site, resulting in enhanced desalination capacity. Besides, since Na^+^ in P‐NMO is adjusted at prismatic sites, it can directly migrate between prismatic sites through an open square path without passing through intermediate sites with lower diffusion barriers, which is the origin of the highest desalination capacity of the P‐NMO.

Na_*x*_CoO_2_ (NCO), composed of the multiple CoO_2_ plate and sodium ion layers, is another research focus in CDI because of its stable and open framework structure for sodium ion diffusion. According to studies,^[^ ^83^, ^84^ ^]^ the P2‐type NCO could have various sodium content with different Na^+^/vacancy structure. As a result, the sodium content could greatly affect the electrical conductivity and the arrangement of sodium vacancies, which ultimately become an important factor for its desalination performance as HCDI electrodes.

Na*_x_*Ti*_n_*O_2_ *_n_*_+1_ (NTO) with layered or tunnel structure has been widely used in SIB relating to its advantages of low cost, low toxicity, and easy preparation. Li et al. reported the synthesis of carbon@Na_4_Ti_9_O_20_ (C@NTO) core–shell nanotubes as a novel electrode for hybrid capacitor deionization (HCDI).^[^ ^54^ ^]^ Owing to the superior insertion desalination ability, the C@NTO‐based HCDI exhibits a high salt removal capacity of 66.14 mg g^−1^. Furthermore, the desalination mechanism was confirmed through CV measurements, which could be expressed as follow (peaks locating at −0.22 and −0.38 V, corresponding to the reaction between Ti^4+^/Ti^3+^)

(4) $$Na_{4}Ti_{9}O_{20}+9Na^{+}+9e^{-}↔Na_{13}Ti_{9}O_{20}$$

**Figure 7:**
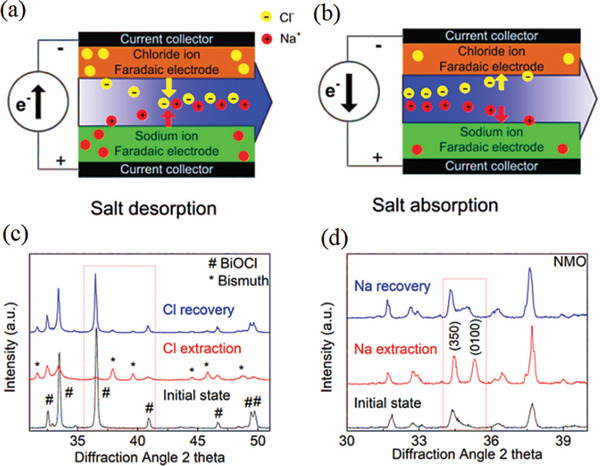
a,b) Schematics of working mechanism of dual‐ions electrochemical deionization device, c) XRD patterns of the initial BiOCl, its state of chloride extraction, and state of chloride recovery. As‐prepared BiOCl (black), chloride extraction (red), the chloride recovery (blue), # BiOCl, *Bismuth, d) XRD patterns of the as‐prepared NMO, its state of sodium extraction, and state of sodium recovery. Reproduced with permission.^[^ ^85^ ^]^ Copyright 2017, The Royal Society of Chemistry.

In addition to solo ions intercalation, Yang et al. proposed a new concept of dual‐ions electrochemical deionization (DEDI) system that utilized NMO and BiOCl as a negative and positive electrode to achieve high‐performance sodium and chloride removal through generate faradaic reaction, as shown in **Figure** **7**a,b.^[^ ^85^ ^]^ The DEDI system delivers a stable and reversible desalination capability of 68.5 mg g^−1^ and a charge efficiency of up to 95.8% at a current density of 100 mA g^−1^, which demonstrates an excellent desalination performance. Furthermore, the XRD tests of the electrodes before and after the desalination process were performed to get a better insight into the destination mechanism of the DEDI, as shown in Figure 7c,d. The diffraction peaks of BiOCl became weak, while the peaks of bismuth metal emerged, indicating that the BiOCl is partially converted to Bi after chloride extraction. Meanwhile, the diffraction peaks of BiOCl reappear after salination, which indicates the recovery of BiOCl. On the other hand, the NMO electrode undergoes a similar process while its diffraction peaks are shifted to small‐angle during desalination process and shifts back during salination, suggesting the reversible intercalation reaction of NMO.

Afterwards, Li et al. developed another type of DEDI system employing Na_1.1_V_3_O_7.9_ (NVO)@reduced graphene oxide (rGO) as a SII electrode and Ag@rGO as chloride ion intercalation electrode,^[^ ^86^ ^]^ which realized an outstanding desalination performance that the salt removal capacity reaches up to 82.2 mg g^−1^ due to the unique band structure of NVO as well as the conductive network structure offered by rGO.

To date, TMOs have been intensively studied as electrode material for CDI‐related applications (including ACDI, HCDI, and DEDI) because of their facial preparation process and structural adjustability. However, two main challenges remain for TMOs‐based CDI systems, the relatively low desalination capacity, and low cycle stability, which have become the bottle‐neck for their practical applications.

### 2.3 Polyanionic Phosphates

Sodium iron pyrophosphate (Na_2_FePO_7_) with triclinic crystal structure constructed by sharing angles between metal polyhedral (FeO_6_ and FeO_5_) and pyrophosphate P_2_O_7_ groups^[^ ^87^, ^88^ ^]^ has received intensive attention in sodium ion storage due to its advantages of abundant sodium ion sites, low manufacturing cost, high thermal (chemical) stability and environmental friendliness. Kim et al. firstly introduced Na_2_FeP_2_O_7_ into HCDI as the sodium removal electrode,^[^ ^89^ ^]^ and acquired a high desalination capacity of 30.2 mg g^−1^ with desalination rate up to 0.081 mg g^−1^ s^−1^.

**Figure 8:**
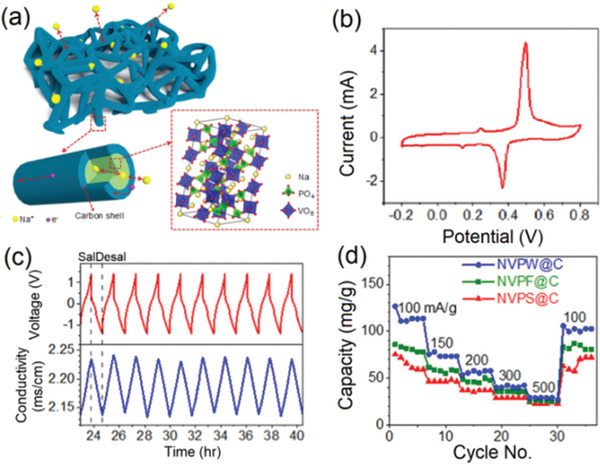
a) Schematics of the NVPW@C ion conductor network and NVP atomic structure, b) CV curve of the NVPW‐AgCl electrode system in 1.0 m NaCl aqueous solution with a scan rate of 1 mV s^−1^, c) voltage vs time and corresponding conductivity vs time during salination and desalination processes at a current density of 100 mA g^−1^, d) salt removal performance of NVP@C‐AgCl EDI devices. Reproduced with permission.^[^ ^57^ ^]^ Copyright 2018, The Royal Society of Chemistry.

As a typical NASICON material, Na_3_V_2_(PO_4_)_3_ (NVP) enjoys high theoretical capacity of 117.6 mAh g^−1^, yet the intrinsic poor conductivity has hindered its sodium storage applications. Recently, great efforts have been devoted to improve the conductivity of NVP by constructing a carbon layer on the surface of NVP. Zhao et al. developed three different type of carbon‐coated NVP (NVP@C) with spherical, flower‐like and linear shaped structures via solvothermal method,^[^ ^57^ ^]^ and further utilized as cathode for DEDI (AgCl as anode). The linear structure of NVP@C forms an ion conductor network realating to the best desalination performance. **Figure** **8**b exhibits the CV curve of NVPW‐AgCl device in 1.0 m NaCl with a scan rate of 1.0 mV s^−1^. There are two distinct characteristic peaks at 0.50 and 0.36 V, which are ascribed to the extraction and reintercalation of sodium ions into the NVP electrode. As predicted, the NVPW@C||AgCl‐based DEDI realizes the high desalination capacity (102.1 mg g^−1^) with outstanding cycling performance (reduced from 124.0 to 98.0 mg g^−1^ after 50 cycles).

In addition to crystalline polyphosphates, amorphous polyanionic phosphates is another potential candidate for HCDI.^[^ ^90^ ^]^ Dai et al. synthesized graphene‐coated mesoporous amorphous FePO_4_ nanospheres (FePO_4_@RGO) through a one‐step hydrothermal method^[^ ^91^ ^]^ for HCDI. The FePO_4_@RGO displays a mesoporous network structure, which could promote the penetration of the electrolyte into the electrode and subsequently facilitates the diffusion of sodium ion. As a result, the desalination capacity and rate of the FePO_4_@RGO‐based HCDI are increased by 60% (50.13–85.94 mg g^−1^) and 180% (0.079–0.24 mg g^−1^ s^−1^), while the energy consumption was reduced by 25% comparing to that of FePO_4_.

### 2.4 Prussian Blue Analogs

The Prussian blue analogs (PBAs), a type of transition metal (iron, manganese, nickel, etc.) ferrous cyanide, has attracted much attention due to its unique open frame structure and large ion channel that allows sodium ion insertion/extraction. The PBAs can often expressed as A*_x_*M[M(CN)_6_]·zH_2_O (0 < *x *≤ 2), where A represents alkali metal ions, such as Na; M refers to transition metals, such as Fe, Co, Mn, etc.^[^ ^92^ ^]^ In 2017, Guo et al. synthesized single crystal FeFe(CN)_6_ (PB) nanoparticles and used as negative electrode (activated carbon as the positive electrode) for HCDI.^[^ ^54^ ^]^ The PB‐based HCDI displays an excellent desalination ability (up to 101.7 mg g^−1^) with ultrafast ion removal rate. In order to further improve the desalination performance, the PB was coupled with reduced graphene oxide to construct a PB@NPG composite. As a result, the PB@NPG provides an improved desalination capacity of 120.0 mg g^−1^. This is attributed to the two‐electron reaction of FeFe(CN)_6_ and the large interstitial sites and tunnels of PB.

**Figure 9:**
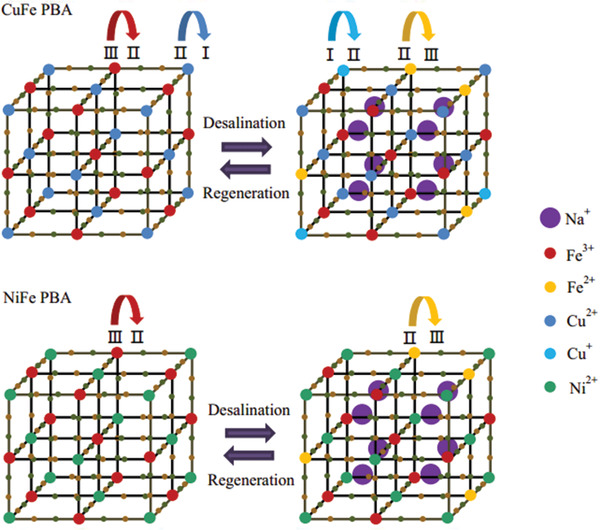
Schematic illustration of desalination mechanism of CuFe@NiFe PBA. Reproduced with permission.^[^ ^93^ ^]^ Copyright 2019, The Royal Society of Chemistry.

Despite the high capacity enabled by bimetallic PBAs, such as CuFe PBA, FeFe PBA, and MnFe PBA, their cycling stability is not satisfied yet. Zhao et al. proposed a feasible stratergy by wrapping PBA in a strong shell (CuFe@NiFe) to improve its structural stability.^[^ ^93^ ^]^ The desalination results suggest that CuFe@NiFe PBA‐based HCDI promoise an improved desalination capacity (54.3–71.8 mg g^−1^) and cycing stability (8% reduction after 50 cycles). The enhanced desliantion performance could be ascribed to the following factors: i) the unique core–shell heterogeneous structure facilitates the ion transfer and subsequently accelerates the sodium ion insertion; ii) the core–shell heterostructure effectively prevents the structural change during repeat ion intercalation and deintercalation and further improve the long term stability of the CuFe@NiFe PBA‐based HCDI. **Figure** **9** shows the desalination and regeneration mechanism of CuFe@NiFe PBA. During the desalination process, the sodium ions are chemically inserted into the electrode, while the Fe^3+^ and Cu^2+^ in the CuFe PBA were reduced to Fe^2+^ and Cu^+^. In the regeneration process, sodium ions were deintercalated from the electrode, while Fe^2+^ and Cu^+^ were oxidized into Fe^3+^ and Cu^2+^. Thus, the overall desalination process is essentially controlled by the redox pair of Fe^3+^/Fe^2+^ and Cu^2+^/Cu^+^.

**Figure 10:**
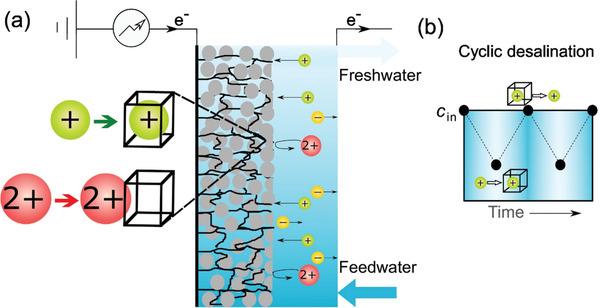
a) Overview of CDI with NiHCF electrodes in monovalent/divalent ion mixtures, b) concentration change in circulation mode. Reproduced with permission.^[^ ^94^ ^]^ Copyright 2020, Elsevier.

PBAs has also been utilized towards the application of selective removal of certain ion species by using the cation hydration radius difference between ions. Singh et al. developed an symmetric two‐compartment CDI, which is composed of two NiHCF electrodes with the same mass.^[^ ^94^ ^]^ It has been found in CDI and other electrochemical technologies that it possess a size‐based preference for cations. In this experiment, the mixed solution contains monovalent ions (Na^+^) and divalent ions (Ca^2+^, Mg^2+^). **Figure** **10**a depicts the operation of intercalated‐compartments of a symmetrical desalination tank. The inset in the figure schematically illiminates the size‐based repulsion of cations by the NiHCF lattice. Figure 10b realizes the concentration change of monovalent ion solution in the cyclic CDI mode, illustrating that the solution concentration is utimately relating to intercalation/de‐intercalation process. It further stands out that the monovalent cations are preferentially adsorbed due to the limitations of NiHCF lattice size. Moreover, it is found that in the case of long‐term selectivity, the NiHCF electrode would preferentially remove monovalent ions (Na^+^) rather than divalent ions (Ca^2+^, Mg^2+^). In practical applications, the preferential removal of Na^+^ is critical to maintain the quality of irrigation water since the high concentrated Na^+^ would have negative impact on soil and plant growth.

Acturally, cations are more important than anions in ion separation because of their higher recovery value, higher toxicity and activity. Among various cations, the removal performance of monovalent cations is generally better than that of divalent or multivalent cations. This is because multivalent cations have complex electrochemical properties and larger hydration radius than monovalent cations. Thus, intercalation materials are still in their infancy in the use of ion separation and ion removal, and more effort is required.

### 2.5 Transition Metal Dichalcogenides

Owing to the excellent electrochemical and mechanical properties, layered 2D TMDs has attracted a lot attentions in the field of energy storage.^[^ ^95^ ^]^ Typically, TMDs enjoyes a layerd structure with one layer of the transition metal atoms is sandwiched between two layers of sulfur atoms. It can be expressed as MX_2_ (where M is the transition metal (i.e., Mo, Ti, V, etc.), and X represents the sulfur group element (S, Se, Te)). Srimuk et al. developed a molybdenum disulfide/carbon nanotube (MoS_2_/CNT) composite material and used as electrode of HCDI.^[^ ^96^ ^]^ The results shows that sodium ions can be inserted between MoS_2_ layers, leading to incensement of the interlayer distance and the change of electronic structure, while the salt removal capacity of 25 mg g^−1^ could be achieved in 500 × 10^−3^ m NaCl concentration.

### 2.6 MXenes

Recently, MXene has rapidly become a major research focus in the energy storage community due to its hydrophilic surface, large interlayer distance, and high electric conductivity.^[^ ^97^ ^]^ Srimuk et al. first introduced Ti_3_C_2_‐MXene into the CDI community electrode,^[^ ^98^ ^]^ yet only 13 mg g^−1^ desalination capacity was achieved, since the serious stacking of the multilayered Ti_3_C_2_‐MXene.

Despite the great improvements achieved for the MXene‐based CDI systems so far, the easily stacked property resulted from the weak van der Waals forces between layers has become the limitation of its further development. To address this issue, Bao et al. synthesized porous MXene by vacuum freeze drying approach.^[^ ^99^ ^]^ By this treatment, the restack of MXene nanosheets can be alleviated. As a result, the accessibility of electrolyte in the pores is improved which favors to increase the overall capacitance. Beyond that, the porous Ti_3_C_2_T*_x_* structure forms 3D network, which provides a multidimensional ion diffusion path for the ions. Subjecting to desalination, the porous Ti_3_C_2_T*_x_* MXene electrode demonstrates the salt removal capacity of 118 mg cm^−3^ (corresponding to 45 mg g^−1^) in 10 000 mg L^−1^ NaCl solution at voltage of 1.2 V).

**Figure 11:**
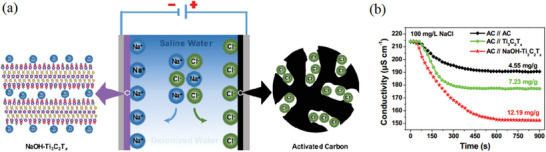
a) The schematics of asymmetric CDI system (AC//NaOH‐Ti_3_C_2_T*_x_*). b) Conductivity profiles of AC//NaOH‐Ti_3_C_2_T*_x_*, AC//Ti_3_C_2_T*_x_*, and AC//AC in 100 mg L^−1^ NaCl solution. Reproduced with permission.^[^ ^100^ ^]^ Copyright 2020, American Chemical Society.

Acturally, MXene owns a highly negatively charged surface of —OH, —O, and —F groups, which may become a cationic selective cathode material reducing the effect of co‐ion impaction. However, HF‐etched MXene suffers from a high ion diffusion barrier and low ion storage capacity due to the small interlayer spacing. In alkaline solution, cations are easily and spontaneously intercalated into Mxene which is favor for expanding the interlayer spacing, leading to activation of the transport of charged ions. Chen et al. assembled an asymmetric CDI system, in which Na^+^ intercalated Ti_3_C_2_T*_x_* (NaOH‐Ti_3_C_2_T*_x_*) and AC is employed as the cation selective cathode and anode, respectively. Most significantly. The NaOH‐Ti_3_C_2_T*_x_* with negatively charged surface groups (—OH, —O, —F) enable to reduce the co‐ion discharge effect.^[^ ^100^ ^]^ Thanks to the reduction of the expulsion of the co‐ion effect and the synergistic effect, the asymmetric CDI system acquires a desalination capacity of 12.19 mg g^−1^ and charging efficiency of 82.6%. Corresponding desalination mechanism is discussed in **Figure** **11**a. After NaOH treatment, the interlayer spacing of Ti_3_C_2_T*_x_* increased from 9.8 to 12.1 Å, which is beneficial to the diffusion and storage of sodium ions between the layers. Figure 11b shows the conductivity distribution of three CDI systems in 100 mg L^−1^ NaCl solution, manifesting the advances of AC ‖NaOH‐Ti_3_C_2_T*_x_* configuration.

**Figure 12:**
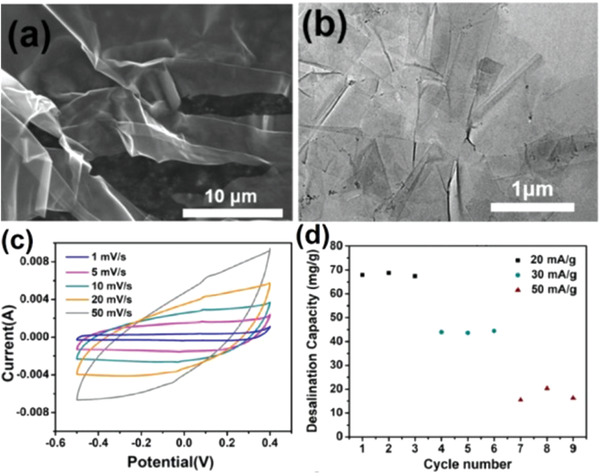
a) SEM images of Ti_3_C_2_T*_x_* after ultrasonic delamination, b) TEM images of Ti_3_C_2_T*_x_*, c) CV curves at different scan rates, d) desalination capacity at different current densities. Reproduced with permission.^[^ ^101^ ^]^ Copyright 2020, Elsevier.

As time goes by, Yu et al. prepared Ti_3_C_2_T*_x_* MXene using LiF/HCl‐etching strategy and further used as the electrode for CDI.^[^ ^101^ ^]^ The results show that Ti_3_C_2_T*_x_* change from multiple layers to few layers or even a single layer after ultrasonic layering, as shown in **Figure** **12**a,b. The CV curve of Ti_3_C_2_T*_x_* electrode presented in Figure 12c demonstrated a distorted rectangle shape, proving the presence of Faraday pseudocapacitance beyond EDL capacitance. Figure 10d shows that the desalination capacity is very stable at any certain current density, suggesting the good cyclic stability of Ti_3_C_2_T*_x_* electrode. Meanwhile, the desalination capacity of the Mxene‐based CDI system is significantly enhanced (up to 68 mg g^−1^) with excellent longterm stability.

## 3 Outlook and Summary

The last 5 years have witnessed the rapid development of SII electrode material for CDI related applications. The salt removal performances of various SII electrode materials are summarized in **Table** **2**. The development of the SII electrode has brought about a major breakthrough in the desalination capacity of CDI related systems due to the superior ion intercalation desalination mechanism. Despite the great advances of intercalation electrodes‐based CDI systems, several obstacles remain: i) Stability. As can be seen from Table 2, the salt removal capacity decay of intercalation electrodes, such as Na_0.71_CoO_2_, α‐MnO_2_/G and ZnFe_2_O_4_, etc., is pretty serious after few cycles. Although great efforts have been devoted to improving the desalination capacity of the electrodes, the cyclic stability of the systems is seldomly studied, which can be a serious problem for its practical applications. In addition, toxic by‐products could be released from the electrode during long‐term operation. Therefore, long‐term test, together with the water quality monitoring should be conducted for all intercalation electrode‐based systems so as to ensure their reliability; ii) Conductivity. Most of the SII electrodes suffer from problem of low conductivity, which would lead to a relatively low desalination capacity. Actually, the desalination capacity of most metal compounds (such as TMO, Na*_x_*TMO_2_) below the respective theoretical capacitance (referring to Table 2) due to its poor conductivity. On the other hand, the poor crystallinity reduces the capacitance either. One of the most available methods in terms of improving the conductivity of the metal compounds is to reconstruct the bulk material into an ultrathin structure, alternatively, the carbon‐metal compound composite should be another solution. For example, Li et al. have demonstrated that the Na_4_Ti_9_O_20_/rGO composite electrode has achieved the desalination capacity of 41.8 mg g^−1^ which is much higher than that of pure Na_4_Ti_9_O_20_ electrode (22.0 mg g^−1^).^[^ ^102^ ^]^ Besides, the electrical conductivity of a metal compound is ultimately depending on its nanostructure, the size of the nanosheets, and the interconnectivity between the nanoparticles. Therefore, more research attentions are needed to improve the cycling stability and the conductivity of the sodium intercalation electrode materials to meet the pratical requirement for desalination. iii) Balance. At present, most intercalation materials are limited to the adsorption of cations, and there are few studies on intercalation materials that adsorb anions (such as MoS_2_ and MXene). As a result, the unbalanced development of cathode and anode materials would hinder the further development of CDI technology. As such, more researches on cathode intercalation materials is needed. At the same time, according to different ion adsorption characteristics, an electrode material database including structure/functional design strategies can be established to comprehensively sort out the characteristics of materials, desalination performance and advantages in different ion adsorption. iv) Mechanism. Currently, great efforts have been dedicated to explore and prepare novel intercalation mateirals aiming to strengthen the desalination performance of CDI, few investigations are focusing on the development of advanced technique to monite the desalination behavior of intercalation electrodes. Fortunately, with the aid of in situ characterization technology, such as in situ XRD, FTIR, and TEM, in‐depth studies are presumbly employed to further understand the structure‐desalination activity relationship of intercalation electrodes.

**Table 2:** Summary of the desalination performance of various electrodes

Electrode materials	Structure	*R*_s_/*R*_ct_	Potential [V]	Current [mA g^−1^]	Concentration [mg L^−1^]	SAC [mg g^−1^]	Stability	Theoretical capacitance [mAh g^−1^]	Ref.
Na_4_Mn_9_O_18_			1.2		580	31.2		40–50	^[^ ^48^ ^]^
FeFe(CN)_6_(PB)				125	1000	120.0	77% after 100 cycles	180	^[^ ^54^ ^]^
Na_4_Ti_9_O_20_		1.72/2.26	1.4		1000	66.14		>200	^[^ ^55^ ^]^
NaTi_2_(PO_4_)_3_	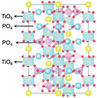			100	2500	105	95% after 100 cycles	133	^[^ ^56^ ^]^
NVP@C	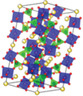			100	1000	102.1	79% after 50 cycles	117.6	^[^ ^57^ ^]^
Na_0.71_CoO_2_	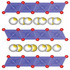	2.6/1.7	1.2		500	34.8	66% after 50 cycles	125	^[^ ^58^ ^]^
Na_0.7_MnO_2_		3.46/22.99	1.2		250	10.7			^[^ ^59^ ^]^
PB/PANI composite				500	10 000	133.9			^[^ ^60^ ^]^
MnHCF			1		1000	19.1	72% after 280 cycles		^[^ ^61^ ^]^
MoS_2_/rGO	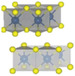		1.4		500	34.2			^[^ ^62^ ^]^
Ti_3_C_2_T*_x_*‐Mxene			1.2		1170	12	94.67% after 50 cycles		^[^ ^63^ ^]^
Anatase TiO_2_	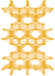			10	500	41.8	over 50 cycles		^[^ ^72^ ^]^
TuMOs	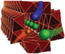		1.2		878	27.8			^[^ ^74^ ^]^
α‐MnO_2_/G	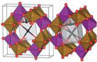	– /2.3	1.2		100	29.5	98% after 4 cycles		^[^ ^75^ ^]^
Fe_2_O_3_@C	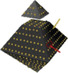	2.2/6.3	1.0		500 (Pb^+^)	830.17	93% after 10 cycles		^[^ ^76^ ^]^
ZnFe_2_O_4_	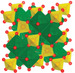			30	5850	136.6	66% after 200 cycles	1000.5	^[^ ^78^ ^]^
NiCo_2_O_4_	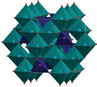	2.65/0.98	1.2		529	44.3	97.5% after 20 cycles	1198	^[^ ^79^ ^]^
P‐NMO	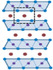		1.0		2925	48	over 30 cycles		^[^ ^82^ ^]^
Na_0.44_MnO_2_	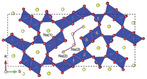			100	760	68.5		540	^[^ ^85^ ^]^
Na_1.1_V_3_O_7.9_@rGO			1.4		2000	82.2		>200	^[^ ^86^ ^]^
Na_2_FePO_7_	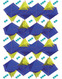		1.2		585	30.2		97	^[^ ^89^ ^]^
FePO_4_@rGO	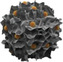			100	2340	85.94	100% after 10 cycles	178	^[^ ^91^ ^]^
CuFe@NiFe Prussian Blue	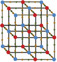			100	2925	71.8	92% after 50 cycles		^[^ ^93^ ^]^
MoS_2_/CNT			0.8		500	25			^[^ ^95^ ^]^
Ti_3_C_2_‐MXene			1.2		292.5	13			^[^ ^96^ ^]^
Porous MXene		1.3/2.6	1.2		10 000	45			^[^ ^99^ ^]^
Ti_3_C_2_T*_x_* MXene	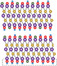	1.52/2.59		20	585	67.7	93% after 30 cycles		^[^ ^101^ ^]^

## Conflict of Interest

The Authors declare no conflict of Interest.
